# Mushroom Poisoning—A 17 Year Retrospective Study at a Level I University Emergency Department in Switzerland

**DOI:** 10.3390/ijerph15122855

**Published:** 2018-12-14

**Authors:** Sarah A. Keller, Jolanta Klukowska-Rötzler, Katharina M. Schenk-Jaeger, Hugo Kupferschmidt, Aristomenis K. Exadaktylos, Beat Lehmann, Evangelia Liakoni

**Affiliations:** 1 Department of Emergency Medicine, Inselspital, University Hospital Bern, University of Bern, 3010 Bern, Switzerland; sarah.keller@students.unibe.ch (S.A.K.); jolanta.klukowska-roetzler@insel.ch (J.K.-R.); aristomenis.exadaktylos@insel.ch (A.K.E.); beat.lehmann@insel.ch (B.L.); 2 National Poisons Information Centre, Tox Info Suisse, Associated Institute of the University of Zurich, 8032 Zurich, Switzerland; katharina.schenk@toxinfo.ch (K.M.S.-J.); hugo.kupferschmidt@toxinfo.ch (H.K.); 3 Clinical Pharmacology and Toxicology, Department of General Internal Medicine, Inselspital, Bern University Hospital, University of Bern, 3010 Bern, Switzerland; 4 Institute of Pharmacology, University of Bern, 3010 Bern, Switzerland

**Keywords:** mushroom poisoning, mushroom toxicity, emergency department

## Abstract

The consequences of mushroom poisoning range from mild, mostly gastrointestinal, disturbances to organ failure or even death. This retrospective study describes presentations related to mushroom poisoning at an emergency department in Bern (Switzerland) from January 2001 to October 2017. Gastrointestinal disturbances were reported in 86% of the 51 cases. The National Poisons Information Centre and mycologists were involved in 69% and 61% of the cases, respectively. Identification of the mushroom type/family was possible in 43% of the cases. The most common mushroom family was Boletaceae (n = 21) and the most common mushrooms *Xerocomus chrysenteron* (n = 7; four being part of a cluster), *Clitocybe nebularis*, *Lepista nuda* and *Lactarius semisanguifluus* (n = 5 each, four being part of a cluster). Poisonous mushrooms included *Amanita phalloides* (n = 3, all analytically confirmed), *Boletus satanas* (n = 3), *Amanita muscaria* (n = 2) and *Amanita pantherina* (n = 2). There were no fatalities and 80% of the patients were discharged within 24 h. Mushroom poisoning does not appear to be a common reason for emergency consultation and most presentations were of minor severity and related to edible species (e.g., due to incorrect processing). Nevertheless, poisonous mushrooms and severe complications were also recorded. Collaboration with a poison centre and/or mycologists is of great importance, especially in high risk cases.

## 1. Introduction

Of the estimated 5000 existing mushroom species, only 200–300 have been established to be safely edible, while 50–100 are known to be poisonous to humans and the toxicity profile of most other species has not been investigated [1,2]. Depending on the species, consumption of poisonous mushrooms can cause various clinical signs and symptoms, ranging from mild, mostly gastrointestinal, disturbances to organ failure and death [2,3,4,5]. Poisonous mushrooms contain a variety of different toxins whose potency is influenced by many extrinsic and intrinsic factors present around the mushroom [6]. Main categories of mushroom toxins include protoplasmic poisons (result in generalized destruction of cells, followed by organ failure), neurotoxins (which cause neurological symptoms such as coma, convulsions and hallucinations), and gastrointestinal irritants (produce nausea, vomiting, abdominal cramping and diarrhoea) [6,7,8]. Patients with early symptoms (typically between thirty minutes and six hours) often have a favorable outcome, while delayed symptoms (i.e., after six hours) are associated with a higher risk of severe complications [3,8,9]. Although mushroom poisoning make up only a small fraction of the total number of emergency consultations for poisoning [10], this poses an important seasonal and regional problem for public health, with reported mortality rates of 8–12% [11,12] and higher rates (up to 15–30%) with mushrooms containing amatoxin [1,2].

In Switzerland, the National Poisons Information Centre *Tox Info Suisse* provides 24-h medical advice nationwide in cases of (suspected) poisoning. In recent decades, there has been a steady growth in mushroom-related calls, with an autumnal peak seen each year [13]. In 2016, 446 (1.4%) of the 32063 documented consultations for poisoning were related to mushroom poisoning (56% in adults, 43% in children and 1% of unknown age) [10]. The most commonly reported family was Boletaceae (6.3%), while 34.9% of the mushrooms were self-harvested and not further identified [14]. In addition to the medical advice offered by *Tox Info Suisse*, the Swiss Mushroom Control Association (*Schweizerische Vereinigung Amtlicher Pilzkontrollorgane, VAPKO*) offers checkpoints for a voluntary assessment of self-harvested mushrooms, in order to check edibility. In 2014, 4504 harvests were checked by the French-speaking part of VAPKO; 37.9% of those contained inedible, 12.5% poisonous and 1.7% lethal mushroom species [15].

Despite possible regional differences, only a study from the French speaking part of Switzerland has investigated the epidemiology and clinical characteristics of patients presenting with mushroom poisoning at an emergency department (ED) in Switzerland [3], and there are no data available from the German speaking part of the country. The aim of this study was to describe the characteristics and management of presentations related to mushroom poisoning at an emergency department in Bern, Switzerland, during a 17 year period.

## 2. Materials and Methods

This was a retrospective single centre study of patients presenting at the ED of Bern University Hospital (the Inselspital) with signs/symptoms related to mushroom exposure or intoxication during a 17 year period (from 1 January 2001 to 31 October 2017). The ED of the Inselspital is both a primary care facility (walk-in patients) and a tertiary referral centre for other hospitals in the region and provides a catchment area for approximately two million people—with about 46,000 (2017) emergencies admissions a year (≥16 years of age).

Patients admitted to the ED are registered in a specific clinical program: The program Qualicare (Qualidoc, Trimbach, Switzerland) was used for the period from 1 January 2001 to 31 May 2012 and a second program—E.care (E.care BVBA, Turnhout, Belgium)—from 1 June 2012 to 31 October 2017. These databases were used to identify all patients presenting with (suspected) mushroom poisoning. Cases were retrieved using a comprehensive full-text search algorithm with mushroom poisoning, related terms and a large number of specific mushroom names, including their German translation, as search terms. Each identified case was reviewed by one of the authors of the study. We included each patient requiring medical evaluation at the ED for mushroom poisoning, regardless of the circumstances of exposure (i.e., accidental or intentional) and the severity of the complaints. We excluded patients with no history of mushroom consumption and cases where the complaints could be clearly attributed to a cause other than mushroom poisoning based on the available final diagnosis in the medical report and the documented evaluation of the physician assessing the patient.

The following parameters were extracted (if available) from the charts of included patients: age, sex, type of transport to the ED (e.g., by ambulance), date of admission and discharge, mushroom species and how they were obtained (e.g., self-harvest, purchased from commercial sources), circumstances of exposure (e.g., accidental, recreational, suicide), symptoms, interval from ingestion to onset of symptoms, laboratory findings (including detection of α-amanitin in urine), consultation of a mycologist or *Tox Info Suisse*, treatment provided, type of discharge from the ED (e.g., inpatient admission, outpatient therapy), length of hospital stay, and outcome (e.g., complications, death). As in previous studies [3,9], we differentiated between early onset of symptoms (i.e., within six hours of ingestion) and delayed onset (i.e., >six hours). Furthermore, we distinguished between number of “clusters” and number of “patients/cases” [16], i.e., patients admitted together at the ED because they consumed the mushrooms jointly were regarded as one cluster, but each patient individually as one patient/case. Data were analyzed descriptively; numerical data are presented as arithmetic mean and standard deviation (SD) and nominal data as proportion (%). Detection of α-amanitin in urine was performed at Zurich University Hospital using enzyme-linked immunoassay (ELISA) until August 2016 and afterwards liquid chromatography- tandem mass spectrometry (LC-MS/MS) [17]. Data were analysed descriptively using Microsoft Excel (Microsoft, Redmond, WA, USA). The study was approved by the local ethics committee (No. 2017-02243).

## 3. Results

**Figure 1:**
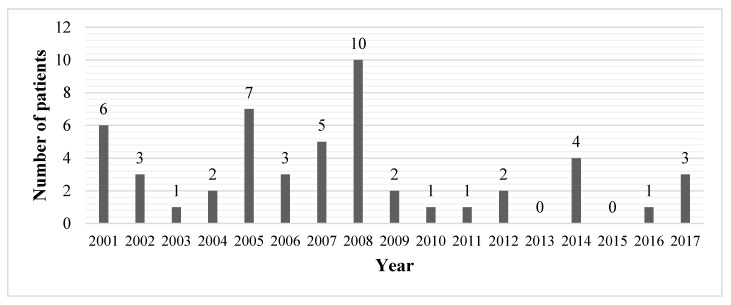
Annual distribution of presentations due to mushroom poisoning (N = 51).

**Figure 2:**
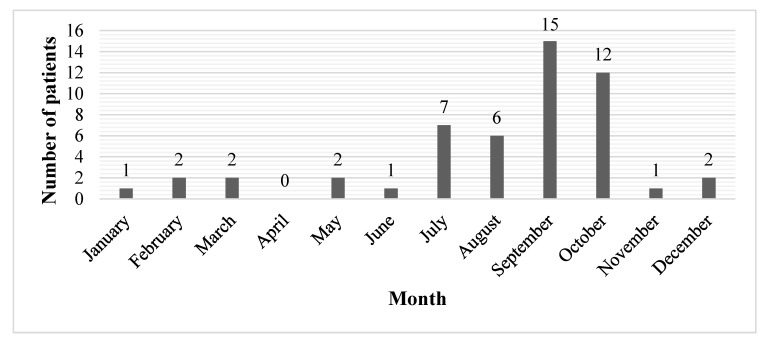
Monthly distribution of presentations for mushroom poisoning (N = 51; bars represent the sum of patients presenting during the same month throughout the study period).

During the study period (1 January 2001 to 31 October 2017), 62 patients were admitted to the ED of Inselspital due to (suspected) mushroom poisoning. Eleven patients were excluded because mushroom consumption could not be confirmed, or the complaints could clearly be attributed to a cause other than mushroom poisoning. Finally, 51 patients were included in our study. The mean (±SD) age of the study population was 48.4 ± 16.9 years (median 48 years), with a range from 15–79 years. Twenty-nine patients (56.9%) were female and 22 (43.1%) male. The annual number of presentations over the study period ranged from zero to ten patients, with a peak in 2008 (Figure 1). The seasonal variation over the period of 17 years showed a peak in autumn (September, October; 27 patients, 52.9%) and in summer (July, August; 13 patients, 25.5%) (Figure 2). 

Table 1 shows the characteristics of the included cases. Forty patients (78.4%) were treated as outpatients and stayed at the ED for less than 24 h. One patient (ID 36) was admitted as inpatient (ward not documented), but was discharged within the first 24 h, and ten patients (19.6%) were hospitalised for more than 24 h (Table 1). In 58.8% (n = 30) of the cases, transport to the ED was organised by the patients themselves, 19.6% (n = 10) by another hospital and the remaining 21.6% (n = 11) by general practitioners, the police, *Tox Info Suisse*, air rescue service or internal allocation. Mycologists were involved in 60.8% (n = 31) of the cases and *Tox Info Suisse* was contacted for 35 patients (68.6%) (Table 1). The exact identification of the mushroom type was possible in 37.3% (n = 19) of the cases. In 13.7% (n = 7), the mushroom species were not definitely identified, in 5.9% (n = 3) only the mushroom family could be determined and in 43.1% (n = 22) the mushroom type remained unspecified/unknown. Table 2 shows the family of the identified and not definitely identified mushrooms, including information about their potential toxicity and other current names [18,19,20,21,22,23,24,25,26,27,28], as well as the reported symptoms and signs and their onset latency in our study.

In 74.5% of the cases (n = 38), the mushrooms were self-harvested and in 94.1% of the cases (n = 48) exposure was accidental. Intentional ingestion included recreational use in two cases (ID 17, 49) and a suicide attempt in one case (ID 33, ingestion of unspecified mushrooms and arum; negative urine analysis for α-amanitin). In most cases, the symptoms of mushroom poisoning were gastrointestinal disturbances; further reported symptoms included neurological, psychiatric, and cardiovascular disorders (Table 3).

**Figure 3:**
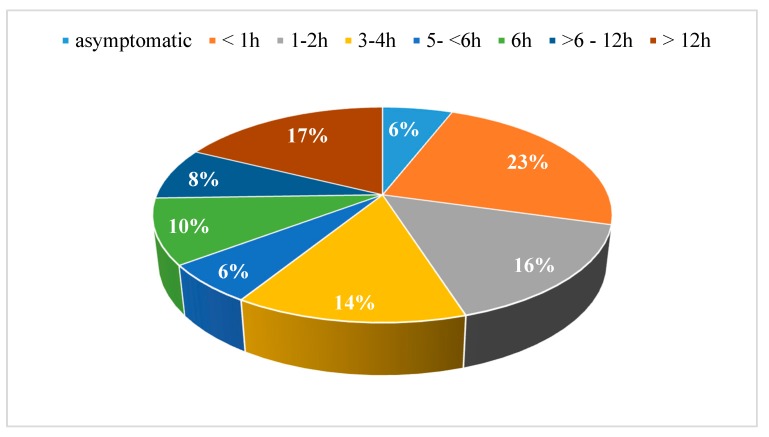
Latency from ingestion to onset of symptoms.

Among the 48 symptomatic patients (94.1%), 35 patients (68.6%) presented with early symptoms (i.e., within the first six hours) and 13 patients (25.5%) with delayed symptoms (i.e., start more than six hours post-ingestion) (Figure 3).

In 52.9% of the cases (n = 27), laboratory abnormalities were found with various deviations from the normal range (Table 1). Elevated transaminases (i.e., alanine aminotransferase (ALAT), aspartate aminotransferase (ASAT)) were found in 11 cases (max. documented ALAT 126 Ul, (mushroom unknown), max. documented ASAT 108 U/l, after ingestion of *Amanita phallloides*); 31.4% of the cases (n = 16) had normal laboratory parameters and in 15.7% (n = 8) no laboratory data were available (Table 1). In 37.3% of the cases (n = 19), the urine was tested for α-amanitin; of these, three (15.8%) were positive (ID 14,19,35) and 16 (84.2%) were negative (ID 1–5,7,12–13,16,20,33–34,36–37,39,45) (Table 1). In the three cases of analytically confirmed *Amanita phalloides* poisoning, the time between ingestion and onset of symptoms ranged from 5.5 to more than 12 h and all three patients were hospitalised (hospitalisation period: five to 33 days). One of these patients (ID 14), developed acute renal failure on the 4th day and elevated liver enzymes with a peak on the 6th day (max. ASAT 108 U/L, ALAT 74 U/L), despite normal laboratory parameters during the first three days (Table 1). The acute renal failure and elevation of the liver enzymes were followed by thrombocytopenia, macrohaematuria, retinal hemorrhage, left side hemiplegia and progressive somnolence. Thrombotic thrombocytopenic purpura due to mushroom intoxication was suspected. After 33 days, the patient was discharged from hospital; no liver or kidney transplantation were needed. Other severe complications included disseminated intravascular coagulation (DIC) and persistent thrombocytopenia in one case (ID 47) after ingestion of *Boletus satanas* (Table 1). After four days, the patient was discharged from hospital with no subsequent complications. Furthermore, unconsciousness was reported by three patients (ID 34,36,42) (Table 1 and Table 3).

In 84.3% of the cases (43 patients), treatment included intravenous fluids (i.e., sodium chloride 0.9%). Symptomatic therapy included metoclopramide (16 patients, 31.4%), butylscopolamine (two patients, 3.9%), meclozine/pyridoxine, domperidone, ondansetron, esomeprazole and paracetamol (one case each, 2%). Twenty-nine patients received activated charcoal (56.9%); 16 patients (31%; ID 2–5,13–15,17,20,32,38–49) were treated with silibinin, 12 patients (24%; ID 2–5,15,20,38–49) with *N*-acetylcysteine, and four (8%, ID 2–5) with penicillin G. Seven patients received no treatment, either because they were asymptomatic, or due to spontaneous resolution of their symptoms. Gastric and jejunal tubes were used in one case due to prolonged diarrhoea and vomiting (Table 1). All patients had favorable outcomes and there were no cases of death or liver failure. Only one patient (ID 14), developed renal failure, but could be discharged from hospital after 33 days without further complications. Table 4 shows the group-intoxications recorded during the study period (14 clusters).

Although the total number of cluster patients was 37, data were not available for ten of those patients who did not consult our ED. Therefore, 27 of the cluster patients are described in our study (Table 1; ID 2–7,12–14,16,21–28,35,37,40,41,45–48,50).

## 4. Discussion

During the study period of 17 years, 51 cases related to mushroom poisoning presented in the ED, with a seasonal peak in autumn and 27 cases as part of a cluster (14 clusters, with only one or some of the persons involved consulting our ED in some cases). In three quarters of the cases, the mushrooms were self-harvested. A mycologist and/or *Tox Info Suisse* were involved in the majority of the cases, and the exact mushroom type or family could be identified in almost half of the cases. In the majority of the cases, the symptoms were gastrointestinal disturbances of minor severity, and the patients were discharged within 24 h after symptomatic therapy was provided. Although there were no cases of death or liver failure, there were three cases of analytically confirmed *Amanita phalloides* poisoning, and, in one case, acute renal failure, thrombotic thrombocytopenic purpura with retinal haemorrhages and left side hemiplegia as complications. Other severe complications included a case of DIC after ingestion of *Boletus satanas*.

Boletaceae was the most commonly identified mushroom family in our study, as in the report from *Tox Info Suisse* [10] and the study from the French part of Switzerland [3], which might indicate that this mushroom is commonly consumed in Switzerland. As in previous studies [2,29], most presentations were related to edible species, which is probably associated with incorrect handling during collection, transport and storage of these mushrooms (e.g., collection in plastic bags, long period of transport at high temperature, long-term storage of mushroom dishes) [29]. Cases with poisonous and potentially life-threatening species were less common and included *Amanita phalloides*, *Boletus satanas*, *Amanita muscaria* and *Amanita pantherina.* Among the main mushroom toxins categories, gastrointestinal irritants were the most common in our study, followed by neurotoxins and protoplasmic poisons. 

A check list of some important points regarding patient history and management in cases of (suspected) mushroom poisoning is contained in the supplement (Table A1, data from [30,31,32,33,34]).

Although not routinely available, analytical methods such as urinary ELISA can be used to detect amatoxin in cases of suspected amatoxin poisoning. This method has good sensitivity (up to 100%), especially if performed within 36 h after ingestion [35,36], although the amatoxin-level does not correlate with the disease severity [37]. In our study, urine was tested for α-amanitin in more than one third of the cases. Of these, the great majority were negative, which might be because of the ingestion of species lacking amatoxin or because the test was performed in the first six hours after ingestion, since detection in urine is only reliable after six hours post-ingestion [36,37], or too late after the meal. The characteristic sequence of the amatoxin-toxidrome with hepatic and multisystem organ failure typically appearing two to six days after mushroom consumption [1,8] was seen in one of the patients in our study with analytically confirmed *Amanita phalloides* poisoning (Table 1, ID 14), who developed nausea and vomiting after a latency of 12 h and liver and kidney function deterioration on the fourth day and after apparent clinical improvement. Coagulopathy, as also seen in this case in our study, is also reported as possible complication in this stage, which spans four to seven days [1]. The other two patients in our study with analytically confirmed *Amanita phalloides* poisoning developed none (ID 35) or only moderate (ID 19) transaminase elevation. Despite this, those patients were hospitalised for several days, most probably to assure regular follow-ups in order to exclude a delay onset hepatic and/or multisystem organ failure. 

Treatment of *Amanita phalloides* poisoning includes administration of activated charcoal for gastrointestinal decontamination and silibinin [37], a milk thistle extract that has been shown to inhibit amatoxin uptake by the OATP1B3 transporters located in hepatocyte membranes [1,38,39] and to lower mortality in cases of amatoxin poisoning [40]. The combination of penicillin G and silibinin is not recommended, since it seems to worsen outcome compared to silibinin alone [41]. In case of hepatotoxicity, recommendations also include administration of *N*-acetylcysteine, similarly to paracetamol intoxication [37,40]. In our study, all three patients with *Amanita phalloides* ingestion received activated charcoal, silibinin and *N*-acetylcysteine. Despite the current recommendation, penicillin G was administered in combination with silibinin in four cases in our study, which might be because older guidelines were applied (all four cases with penicillin G administration were from 2001). Furthermore, since some patients with suspected poisoning received silibinin as precautionary measure while awaiting the analytical results, an in-house test could contribute to avoiding unnecessary administration of drugs and reducing length of hospital stay.

Similarly to other studies [3,4,29], in the majority of our cases the symptoms began with a latency of less than six hours, which is usually associated with a favourable outcome, since mushrooms that only cause acute toxicity are rarely life-threatening [3,8,9]. However, early symptoms do not exclude ingestion of potentially dangerous mushrooms, since patients often ingest more than one type of mushroom and even amatoxin-containing mushrooms can (rarely) cause symptoms before six hours [31,34]. This is also represented in our findings, as one of three patients with analytically confirmed amatoxin poisoning presented with a latency of less than 6 h (Table 1, ID 19). In case of amatoxin-containing mushroom poisoning, earlier onset of gastroenteritis after consumption may correlate with more severe hepatotoxicity [42].

Identification of the specific mushroom(s) involved can contribute to appropriate diagnostic and treatment. In cases of mushroom poisoning, the exact mushroom species is successfully identified in the range of 5–27% of patients [3,31], with only one study [16] reporting higher rates of almost 90%. In our study, we were able to identify the exact mushroom type or family in almost half of the cases, a rate higher than most of the reported rates in the literature, most probably due to the close contact between the Inselspital and *Tox Info Suisse/*mycologists. The authors of the study in Italy [16] also report that the high identification rate was due to the available mycologist service in cases of suspected mushroom poisoning, while, in the study from the French speaking part of Switzerland [3], a mycologist was contacted in only one third of the cases. These findings highlight the importance of collaboration between EDs and the local poison control centres and/or mycologists, in order to enable mushroom identification and professional consultation, especially in cases of high risk.

Similarly to our findings, analysis of mushroom poisoning data from *Tox Info Suisse* also shows marked seasonal fluctuation [2] and most published cases of mushroom poisoning are recorded in the autumn (September and October) and late summer (July and August) [2,3,11,13,16,29]. This autumnal peak is most probably associated with the levels of rainfall, temperature and humidity during this period in Switzerland, which facilitates the growth of mushrooms and is also part of the wild mushroom season (May–October) [2].

The limitations of our study include the retrospective design. Although we aimed to identify all mushroom poisoning cases during the study period using the extensive search of our emergency unit specific software, it is possible that some cases were missed, e.g., if a patient was falsely diagnosed or did not mention the mushroom consumption, or that in some cases the symptoms were attributable to another cause that remained unrecognized despite thorough diagnostic at the ED. Moreover, although it is to assume that the patients remained free of symptoms if there was no re-presentation following their discharge, no information was available regarding their further outcome after leaving the ED. It is also possible that in some cases the treatment did not contribute to the clinical improvement (e.g., when administered as precautionary measure while awaiting the analytical results). Thus, it is not possible to provide an accurate evaluation of the undertaken therapeutic measures, Furthermore, it is possible that the diagnosis of mushroom poisoning was made after discharge from the ED or that patients from peripheral hospitals were directly admitted to a ward, thus resulting in cases that could not be found in our ED database, and it is also possible that some patients developed symptoms or complications after being discharged. Moreover, we cannot report in how many cases identification or exclusion of amatoxin-containing mushrooms was provided by the *Tox Info Suisse/*mycologist before the analytical results were available, since the exact time a mushroom specialist was contacted is not known. As a result, it is also not possible to describe how the patient management was adjusted (e.g., stopped or intensified) based on the mushroom specialist consultation in each case. Finally, our data may not be generalised to other regions in Switzerland, *inter alia* due to differences in local weather. The main strengths of our study include its long duration of 17 years. In addition, we frequently consulted a mycologist and *Tox Info Suisse* and this contributed to our high rate of identification of the mushrooms.

## 5. Conclusions

Based on our findings, ED consultations due to mushroom poisoning do not seem to be very common and mostly include mild symptoms associated with gastrointestinal irritants, usually with favorable outcome. However, although less common, cases of poisonings with potential lethal mushrooms and severe complications were also recorded. Collaboration with the local poison centre and a mycologist is of great importance for the identification of the mushroom(s) involved and decisions on patient management, especially in cases of high risk. The data of our study can contribute to the development of preventive strategies, such as medical personnel training and information material for the public (e.g., using websites or other media) on the correct handling of and potentially life-threatening mushrooms, especially during the high risk season for mushroom poisoning.

## Figures and Tables

**Table 1:** Characteristics of cases presenting due to (suspected) mushroom poisoning, 2001–2017 (N = 51).

Patient ID	Age (Years)	Sex	Latency to Onset of Symptoms	Mushroom Species	Type of Acquisition	Consumed Cooked or Raw	Consultation with a Mycologist or *Tox Info Suisse*	Symptoms/Signs, and Complications	Therapy	Abnormal Laboratory Findings (on Presentation, except Mentioned Otherwise)	Length of Hospital Stay and Transfer to Other Unit
**1**	62	M	>12 h	*Boletus edulis, Macrolepiota procera, Cantharellus cibarius* (not definitely identified)	self-harvested	not known	both	diarrhoea, abdominal pain, vertigo, sweating	activated charcoal	normal findings, alpha-amanitin negative	<24 h, discharged from ED
**2**	15	W	6 h	unknown	self-harvested	not known	both	nausea, vomiting	intravenous fluids, activated charcoal, *N*-acetylcysteine, silibinin, penicillin G	sodium 128 mmol/L, WBC 17.1 G/L, alpha-amanitin negative	<24 h, discharged from ED
**3**	22	M	6 h	unknown	self-harvested	not known	both	nausea, vomiting, abdominal pain	intravenous fluids, activated charcoal, *N*-acetylcysteine, silibinin, penicillin G	WBC 18.1 G/L, alpha-amanitin negative	<24 h, discharged from ED
**4**	48	M	5 h	unknown	self-harvested	not known	both	nausea, vomiting, diarrhoea, abdominal pain	intravenous fluids, activated charcoal, *N*-acetylcysteine, silibinin, penicillin G	creatinine 106 µmol/L, CRP 8 mg/L, bilirubin total 27 µmol/L, alpha-amanitin negative	<24 h, discharged from ED
**5**	46	W	6 h	unknown	self-harvested	not known	both	nausea, vomiting, abdominal pain	intravenous fluids, activated charcoal, *N*-acetylcysteine, silibinin, penicillin G	WBC 17.1 G/L, alpha-amanitin negative	<24 h, discharged from ED
**6**	64	M	4 h	Boletaceae edible	commercial sources	not known	mycologist	nausea, vomiting	intravenous fluids, metoclopramide	ASAT 43 U/L, LDH 527 U/L, WBC 12.7 G/L	<24 h, discharged from ED
**7**	60	W	4 h	Boletaceae edible	commercial sources	not known	mycologist	nausea, vomiting	intravenous fluids, meclizine/pyridoxine, esomeprazole, domperidone	unknown, alpha-amanitin negative	<24 h, discharged from ED
**8**	27	W	0.2 h	unknown	self-harvested	cooked	no	nausea, feeling of faintness, paraesthesia	no therapy	unknown	<24 h, discharged from ED
**9**	36	W	4.5 h	unknown	commercial sources	cooked	*Tox Info Suisse*	nausea, vomiting, diarrhoea, abdominal pain	intravenous fluids, metoclopramide, butyl scopolamine	normal findings	<24 h, discharged from ED
**10**	34	W	2 h	*Russula xerampelina* (not definitely identified)	self-harvested	not known	*Tox Info Suisse*	nausea, vomiting, diarrhoea, abdominal pain	intravenous fluids, paracetamol	ASAT 58 U/L, ALAT 85 U/L, AP 174 U/L, GGT 160 U/L, LDH 530 U/L, CRP 48 mg/L ¶	<24 h, discharged from ED
**11**	30	W	>6 h	*Agaricus edible* (not definitely identified)	commercial sources	not known	no	nausea, vomiting, abdominal pain, hypaesthesia	intravenous fluids, metoclopramide, ondansetrone	normal findings	<24 h, discharged from ED
**12**	60	W	0.5 h	unknown	self-harvested	not known	mycologist	nausea, abdominal pain, dry mouth	intravenous fluids, activated charcoal, silibinin	normal findings, alpha-amanitin negative	<24 h, discharged from ED
**13**	60	M	0.5 h	unknown	self-harvested	not known	mycologist	nausea, abdominal pain, dry mouth	intravenous fluids, activated charcoal, silibinin	normal findings, alpha-amanitin negative	<24 h, discharged from ED
**14 ∇**	67	W	12 h	*Amanita phalloides*	self-harvested	not known	both	prolonged nausea and vomiting: on the 4th day acute renal failure, macrohaematuria, retinal haemorrhages, hemiplegia, progressive somnolence, suspected thrombotic thrombocytopenic purpura	intravenous fluids, activated charcoal, *N*-acetylcysteine, silibinin, metoclopramide	initially normal; on the 4th day increase in liver enzymes (peak on the 6th day; ASAT 108 U/L, ALAT 74 U/L) and creatinine (peak on the 11th day; 398 μmol/L), thrombocytopenia (nadir of 23 G/L on the 9th day); alpha-amanitin positive	792 h, internal medicine, abdominal surgery, intensive unit care, nephrology
**15**	42	M	>12 h	unknown	self-harvested	not known	no	nausea, diarrhoea	no therapy	unknown	<24 h, discharged from ED
**16 ∇**	48	W	>12 h	unknown	self-harvested	cooked	both	nausea, vomiting, diarrhoea, vertigo, fatigue	intravenous fluids, silibinin	ASAT 43 U/L, ALAT 61 U/L, AP 106U/L, GGT 118 U/L, bilirubin total 22 µmol/L ¶,# alpha-amanitin urinalysis negative	84 h, internal medicine
**17**	23	M	6 h	*Amanita muscaria* (not definitely identified)	commercial sources	not known	*Tox Info Suisse*	asymptomatic	no therapy	sodium 148 mmol/L	<24 h, discharged from ED
**18**	70	W	0.2 h	*Lepista nuda, Hypholoma fasciculaea or laterium*	commercial sources (Lepista), unknown (Hypholoma)	not known	both	nausea, abdominal pain	intravenous fluids, metoclopramide	bilirubin total 17 µmol/L	<24 h, discharged from ED
**19 ∇**	74	M	5.5 h	*Amanita phalloides*	self-harvested	cooked	both	nausea, vomiting, diarrhoea, vertigo, cramps	intravenous fluids, activated charcoal, *N*-acetylcysteine, silibinin	ASAT 52 U/L, ALAT 42 U/L, GGT 68 U/L (peak on the 6th day; 79 U/L), creatinine 119 µmol/L, glomerular filtration rate (GFR) 52 mL/min, CRP 7 mg/L, INR 3.8, bilirubin total 17 µmol/L (peak on the 6th day; 38 µmol/L),, alpha-amanitin positive	120 h, intensive care
**20**	59	M	5 h	unknown	self-harvested	cooked	*Tox Info Suisse*	nausea, diarrhoea, abdominal pain, hallucinations	intravenous fluids	potassium 3.3 mmol/L, CRP 7 mg/L, alpha-amanitin negative	<24 h, discharged from ED
**21**	47	W	2 h	*Boletus satanas*	self-harvested	cooked	*Tox Info Suisse*	nausea, vomiting, diarrhoea, abdominal pain, headache, chills	intravenous fluids, metoclopramide, butylscopolamine, esomeprazole	potassium 3.3 mmol/L	<24 h, discharged from ED
**22**	36	W	1.5 h	*Boletus satanas*	self-harvested	raw	both	nausea, vomiting, diarrhoea	intravenous fluids, activated charcoal, metoclopramide	potassium 3.3 mmol/L	<24 h, discharged from ED
**23**	73	W	n.a.	*Clitocybe nebularis, Xerocomus chrysenteron, X. badius, Lepista nuda, Lactarius semisangui fluus, Russula variata, Amanita rubescens, Laccaria amethystina, Craterellus cornuco pioides, Pseudohyd num gelatinosum*	self-harvested	cooked	both	asymptomatic	intravenous fluids, activated charcoal	unknown	<24 h, discharged from ED
**24 ∇**	51	W	0.5 h	same as patient ID 23	self-harvested	cooked	both	nausea, diarrhoea, thoracic pain	intravenous fluids, activated charcoal	normal laboratory findings	55 h, cardiology
**25**	71	M	n.a.	same as patient ID 23	self-harvested	cooked	both	asymptomatic	intravenous fluids, activated charcoal	unknown	<24 h, discharged from ED
**26**	58	M	n.a.	same as patient ID 23	self-harvested	cooked	both	asymptomatic	intravenous fluids, activated charcoal	unknown	<24 h, discharged from ED
**27**	71	M	0.4 h	*Armillaria mellea, Xerocomus chrysenteron, Chlorophyl lum rachodes*	self-harvested	cooked	both	nausea, vertigo, fatigue, confusion, sweating	intravenous fluids, activated charcoal, metoclopramide	ASAT 39 U/L, GGT 44 U/L, LDH 621 U/L, INR 2.15	<24 h, discharged from ED
**28**	69	W	0.4 h	*Armillaria mellea, Xerocomus chrysenteron, Chlorophyl lum rachodes*	self-harvested	cooked	both	nausea, vomiting, vertigo	intravenous fluids, activated charcoal, metoclopramide	potassium 3.3 mmol/L,	<24 h, discharged from ED
**29**	33	W	>12 h	*Clitocybe nebularis*, Boletaceae	self-harvested	raw	both	nausea, diarrhoea, headache, dry mouth	intravenous fluids, activated charcoal, silibinin	normal laboratory findings	<24 h, discharged from ED
**30**	49	W	6 h	unknown	commercial sources	cooked	*Tox Info Suisse*	nausea, vomiting, diarrhoea, abdominal pain	intravenous fluids, metoclopramide	normal laboratory findings	<24 h, discharged from ED
**31**	73	W	1 h	unknown	self-harvested	raw	no	nausea, vomiting, diarrhoea	intravenous fluids, metoclopramide	LDH 513 U/L, creatinine 95 µmol/L, GFR 50 mL/min	<24 h, discharged from ED
**32**	36	W	>12 h	unknown	restaurant meal	cooked	no	nausea, abdominal pain	intravenous fluids, metoclopramide	ALAT 102 U/L, GGT 90 U/L, bilirubin total 49 µmol/L	<24 h, discharged from ED
**33 ∇**	58	W	0.5 h	unknown	self-harvested	raw	*Tox Info Suisse*	salivation	intravenous fluids, activated charcoal, *N*-acetylcysteine, silibinin	GGT 95 U/L, alpha-amanitin negative	72 h, intensive care
**34 ∇**	79	M	4.5 h	unknown	self-harvested	cooked	both	nausea, vomiting, vertigo, unconsciousness	intravenous fluids, activated charcoal, *N*-acetylcysteine, silibinin	potassium 5.7 mmol/L, creatinine 88 µmol/L, GFR 87 mL/min, WBC 11.9 G/L, alpha-amanitin negative	120 h, intensive care
**35 ∇**	67	M	>12 h	*Amanita phalloides*	self-harvested	not known	mycologist	nausea, vomiting, diarrhoea	intravenous fluids, activated charcoal, *N*-acetylcysteine, silibinin, gastric and jejunal tube	CRP 30 mg/L, bilirubin total 37 µmol/L, WBC 13.1 G/L, INR 1.3, alpha-amanitin positive	216 h, intensive care
**36**	53	W	>6 h	unknown	self-harvested	cooked	*Tox Info Suisse*	nausea, vomiting, diarrhoea, vertigo, unconsciousness	intravenous fluids, activated charcoal, *N*-acetylcysteine, silibinin	normal findings, alpha-amanitin negative	<24 h, ward not documented
**37 ∇**	65	W	0.3 h	Mushrooms with gills, *Lactarius semisanguifluus*, Boletaceae	self-harvested	not known	both	nausea, vomiting, diarrhoea, tachycardia, sweating	intravenous fluids, activated charcoal, *N*-acetylcysteine, silibinin	ASAT 41 U/L, ALAT 40 U/L, GGT 95 U/L, LDH 508 U/L, creatinine 79 mmol/L, GFR 68 mL/min, CRP 9 mg/L, WBC 18.6 G/L, haemoglobin 117 g/L, alpha-amanitin negative	72 h, gastroenterology ward
**38 ∇**	41	W	7 h	unknown	self-harvested	not known	*Tox Info Suisse*	nausea, vomiting, paraesthesia	intravenous fluids, activated charcoal, *N*-acetylcysteine, silibinin	normal findings	30 h, intensive care unit
**39**	30	W	4 h	*Macrolepiota procera*	self-harvested	not known	both	nausea, vomiting, diarrhoea, abdominal pain	intravenous fluids, activated charcoal	normal findings, alpha-amanitin negative	<24 h, discharged from ED
**40**	54	M	0.8 h	*Amanita pantherina*	self-harvested	cooked	mycologist	nausea, vomiting, abdominal pain, fatigue, mild visual disturbances, agitation, psychological excitation, tachycardia	intravenous fluids, activated charcoal	ASAT 40 U/L, creatinine 104 µmol/L	<24 h, discharged from ED
**41**	54	W	0.8 h	*Amanita pantherina*	self-harvested	cooked	mycologist	abdominal pain, fatigue, agitation, mild visual disturbances	intravenous fluids, activated charcoal	normal findings	<24 h, discharged from ED
**42**	19	M	>12 h	*Psilocybe*	purchased on the street	raw	no	vertigo, feeling of faintness, unconsciousness, dyspnoea	intravenous fluids	normal findings	<24 h, discharged from ED
**43**	46	M	0.3 h	*Boletus edulis* (not definitely identified)	restaurant meal	cooked	no	nausea, abdominal pain	intravenous fluids, metoclopramide	normal findings	<24 h, discharged from ED
**44**	53	W	7 h	*Boletus edulis* (not definitely identified)	commercial sources	cooked	no	nausea, diarrhoea, headache, fever	intravenous fluids	normal findings	<24 h, discharged from ED
**45**	43	W	3 h	unknown	self-harvested	cooked	both	nausea, vomiting, diarrhoea	intravenous fluids, activated charcoal, metoclopramide	normal findings, alpha-amanitin negative	<24 h, discharged from ED
**46**	48	M	1 h	unknown	self-harvested	cooked	both	nausea, vomiting, diarrhoea	intravenous fluids, activated charcoal, metoclopramide	GGT 52 U/L	<24 h, discharged from ED
**47 ∇**	40	M	1.5 h	*Boletus satanas*	self-harvested	raw	both	nausea, vomiting, diarrhoea, tachycardia, hypotension, fever, suspected disseminated intravascular coagulation (DIC)	intravenous fluids, activated charcoal, metoclopramide	ASAT 36 U/L, INR 1.32, persistent thrombocytopenia: nadir on the 3th day 102 G/L	105 h, internal medicine ward
**48**	24	M	>12 h	unknown	self-harvested	cooked	*Tox Info Suisse*	headache	no therapy	ASAT 38 U/L, ALAT 126 U/L	<24 h, discharged from ED
**49**	23	M	1 h	*Amanita muscaria* (not definitely identified)	purchased on the street	raw	no	nausea, vomiting, fatigue, aggression	no therapy	WBC 14.4 G/L	<24 h, discharged from ED
**50**	28	W	4 h	*Lycoperdales, Xerocomus chrysenteron*	self-harvested	raw	both	nausea, vomiting, diarrhoea, chills	no therapy	unknown	<24 h, discharged from ED
**51**	26	M	1 h	unknown psycho-active mushrooms	purchased on the street	raw	*Tox Info Suisse*	nausea, vomiting, extrapyramidal symptoms, panic, sweating	no therapy	unknown	<24 h, discharged from ED

∇ Hospitalisation > 24 h; ¶ Liver enzymes and cholestasis parameters elevated prior to mushroom exposure; # Laboratory findings during the rest of the hospitalisation not available; n.a.: not applicable; W: woman; M: man; Reference range: aspartate aminotransferase (ASAT) <35 U/L; alanine aminotransferase (ALAT) <35 U/L, alkaline phosphatase (AP) 35–104 U/L; gamma-glutamyl transpeptidase (GGT) <40 U/L; bilirubin total <17 µmol/L; lactate dehydrogenase (LDH) <480 U/L; C-reactive protein (CRP) <5 mg/L; haemoglobin 135–168 g/L; white blood cell (WBC) 3.00–10.5 G/L; international normalised ratio (INR) 0.7–1.2; sodium 132–146 mmol/L, potassium 3.5–4.5 mmol/L.

**Table 2:** Family [18,19,20,21,22,23,24,25,26,27,28], symptoms/signs and interval from ingestion to symptoms of identified and not definitely identified mushrooms (n = number of cases; more than one mushroom involved in some cases).

Family	Species	n	Comment	Symptoms and Signs	Interval from Ingestion to Symptoms
Agaricaceae (n = 5)	*Agaricus bisporus*	1	Edible (in Switzerland sold only from cultivated mycelium)	nausea, vomiting, abdominal pain, hypesthesia	>6 h
	*Chlorophyllum rachodes*	2	Edible (in Switzerland)	nausea, vomiting, vertigo, confusion, sweating, fatigue	0.4 h
	*Macrolepiota procera*	2	Edible	nausea, vomiting, diarrhea, abdominal pain, vertigo, sweating	4–>12 h
Amanitaceae (n = 11)	*Amanita muscaria*	2	Poisonous	nausea, vomiting, aggression, fatigue	1–6 h
	*Amanita pantherina*	2	Poisonous	nausea, vomiting, abdominal pain, agitation, psychological excitation, visual disturbances, tachycardia, fatigue	0.75 h
	*Amanita phalloides*	3	Poisonous	nausea, vomiting, diarrhea, vertigo, cramps, hemiplegia, somnolence, acute renal failure, macroheamaturia, retinal hemorrhages, thrombotic thrombocytopenic purpura	5.5–>12 h
	*Amanita rubescens*	4	Edible	nausea, diarrhea, thoracic pain, asymptomatic	0.5 h
Boletaceae (n = 21)	*n.a.*	4	Not further identified Edible, non-edible, poisonous depending on the species	nausea, vomiting, diarrhea, headache, dry mouth, sweating, tachycardia	0.3–>12 h
	*Boletus edulis*	3	Edible	nausea, diarrhea, abdominal pain, vertigo, sweating, headache, fever	0.3–>12 h
	*Boletus satanas*	3	Poisonous Current name: *Rubroboletus satanas*	nausea, vomiting, diarrhea, abdominal pain, headache, tachycardia, hypotension, chills, fever, suspected DIC	1.5–2 h
	*Xerocomus chrysenteron*	7	Edible Current name: *Xerocomellus chrysenteron*	nausea, vomiting, diarrhea, vertigo, confusion, sweating, thoracic pain, fatigue, chills, asymptomatic	0.4–4 h
	*Xerocomus badius*	4	Edible Current name: *Imleria badia*	nausea, diarrhea, thoracic pain, asymptomatic	0.5 h
Cantharellaceae (n = 5)	*Cantharellus cibarius*	1	Edible	diarrhea, abdominal pain, vertigo, sweating	>12 h
	*Craterellus cornucopioides*	4	Edible	nausea, diarrhea, thoracic pain, asymptomatic	0.5 h
Hydnangiaceae (n = 4)	*Laccaria amethystina*	4	Edible	nausea, diarrhea, thoracic pain, asymptomatic	0.5 h
Incertae sedis (n = 4)	*Pseudohydnum gelatinosum*	4	Edible	nausea, diarrhea, thoracic pain, asymptomatic	0.5 h
Physalacriaceae (n = 2)	*Armillaria mellea*	2	Edible but has to be pre-cooked in boiling water	nausea, vomiting, vertigo, confusion, sweating, fatigue	0.4 h
Russulaceae (n = 10)	*Russula variata*	4	Edible Current name: *Russula cyanoxantha*	nausea, diarrhea, thoracic pain, asymptomatic	0.5 h
	*Russula xerampelina*	1	Edible	nausea, vomiting, diarrhea, abdominal pain	2 h
	*Lactarius semisanguifluus*	5	Edible	nausea, vomiting, diarrhea, sweating, thoracic pain, tachycardia, asymptomatic	0.3–0.5 h
Strophariaceae (n = 1)	*Hypholoma fasciculaea or laterium*	1	Poisonous	nausea, abdominal pain	0.2 h
Tricholomataceae (n = 10)	*Clitocybe nebularis*	5	Edible in Switzerland but has to be prepared in boiling water Considered non-edible in Germany	nausea, diarrhea, headache, dry mouth, thoracic pain, asymptomatic	0.5–>12 h
	*Lepista nuda*	5	Edible	nausea, abdominal pain, diarrhea, thoracic pain, asymptomatic	0.2–0.5 h
n.a.	*Lycoperdales*	1	Outdated order Edible and non-edible	nausea, vomiting, diarrhea, chills	4 h

n.a.: not applicable.

**Table 3:** Clinical characteristics of mushroom poisoning (N = 51).

	n (%)
**Gastrointestinal**	44 (86.3)
nausea	42 (82.4)
vomiting	30 (58.8)
diarrhoea	23 (45.1)
abdominal pain	18 (35.3)
**Neurological/Psychiatric**	23 (45.1)
vertigo	8 (15.7)
agitation/aggression/panic/psychological excitation	5 (9.8)
headache	4 (7.8)
paraesthesia/hypaesthesia	3 (5.9)
unconsciousness	3 (5.9)
feeling of faintness	2 (3.9)
mild visual disturbances	2 (3.9)
confusion	1 (2)
extrapyramidal symptoms	1 (2)
hemiplegia	1 (2)
spasms	1 (2)
hallucinations	1 (2)
cholinergic symptoms (e.g., sweating, salivation)	5 (9.8)
anticholinergic symptoms (e.g., dry mouth)	3 (5.9)
**Cardiovascular**	5 (9.8)
tachycardia	3 (5.9)
thoracic pain	1 (2)
hypotension	1 (2)
dyspnoea	1 (2)
**Miscellaneous/other**	10 (19.6)
fatigue	5 (9.8)
chills/fever	4 (7.8)
renal failure	1 (2)
(suspected) thrombotic thrombocytopenic purpura	1 (2)
(suspected) disseminated intravascular coagulation (DIC)	1 (2)

**Table 4:** Cases of group intoxication (clusters).

Cluster Size (Number of Persons Involved)	Patient ID	Mushroom Species	Symptoms and Signs	Interval from Ingestion to Symptoms	Comment
4	2–5	unknown	nausea (n = 4), vomiting (n = 4), abdominal pain (n = 3), diarrhoea (n = 1)	5–6 h	
2	6–7	*Boletaceae*	nausea (n = 2), vomiting (n = 2)	4 h	
2	12–13	unknown	nausea (n = 2), abdominal pain (n = 2), dry mouth (n = 2)	0.5 h	
2	14	*Amanita phalloides*	nausea (n = 1), vomiting (n = 1), acute renal failure (n = 1), macrohaematuria (n = 1), retinal haemorrhages (n = 1), hemiplegia (n = 1), progressive somnolence, (n = 1), suspected thrombotic thrombocytopenic purpura (n = 1)	12 h	Only one patient of this cluster consulted our ED
2	16, 48	unknown	nausea (n = 1), vomiting (n = 1), diarrhoea (n = 1), fatigue (n = 1), vertigo (n = 1), headache (n = 1)	>12 h	
2	21	*Boletus satanas*	nausea (n = 1), vomiting (n = 1), abdominal pain (n = 1), diarrhoea (n = 1), chills (n = 1), headache (n = 1)	2 h	Only one patient of this cluster consulted our ED
3	22, 47	*Boletus satanas*	nausea (n = 2), vomiting (n = 2), diarrhoea (n = 2), fever (n = 1), tachycardia (n = 1), hypotension (n = 1), suspected DIC (n = 1)	1.5 h	Only two patients of this cluster consulted our ED
4	23–26	*Xerocomus chrysenteron, X. badius, Lepista nuda, Clitocybe nebularis, Lactarius semisanguifluus, Russula variata, Amanita rubescens, Laccaria amethystina, Craterellus cornucopioides, Pseudohydnum gelatinosum*	nausea (n = 1), diarrhoea (n = 1), thoracic pain (n = 1)	0.5 h	Only one patient had symptoms, the other three were asymptomatic
2	27, 28	*Xerocomus chrysenteron, Chlorophyllum rachodes, Armillaria mellea*	nausea (n = 2), vertigo (n = 2), vomiting (n = 1), sweating (n = 1), fatigue (n = 1), confusion (n = 1)	0.4 h	
2	35	*Amanita phalloides*	nausea (n = 1), vomiting (n = 1), diarrhoea (n = 1)	>12 h	Only one patient of this cluster consulted our ED
2	37	Mushrooms with gills, *Lactarius semisanguifluus, Boletaceae*	nausea (n = 1), vomiting (n = 1), diarrhoea (n = 1), sweating (n = 1), tachycardia (n = 1)	0.3 h	Only one patient of this cluster consulted our ED
4	40, 41	*Amanita pantherina*	abdominal pain (n = 2), fatigue (n = 2), agitation (=2), mild visual disturbances (n = 2), nausea (n = 1), vomiting (n = 1), psychological excitation (n = 1), tachycardia (n = 1)	0.8 h	Two patients of this cluster visited another ED
3	45, 46	unknown	nausea (n = 2), vomiting (n = 2), diarrhoea (n = 2)	1–3 h	Only two patients of this cluster consulted our ED
3	50	*Lycoperdales, Xerocomus chrysenteron*	nausea (n = 2), vomiting (n = 2), diarrhoea (n = 2), chills (n = 1), asymptomatic (n = 1)	4–12 h	Only one patient of this cluster consulted our ED

## Abbreviations

ALAT	alanine aminotransferase
AP	alkaline phosphatase
ASAT	aspartate aminotransferase
CRP	C-reactive protein
DIC	disseminated intravascular coagulation
ED	emergency department
ELISA	enzyme-linked immunoassay
GFR	glomerular filtration rate
GGT	gamma-glutamyl transpeptidase
h	hour(s)
INR	international normalized ratio
LC-MS/MS	liquid chromatography-tandem mass spectrometry
LDH	lactate dehydrogenase
M	man
n.a.	not applicable
OATP	organic anion transporting polypeptides
SD	standard deviation
W	woman
WBC	white blood cell

## Appendix A

**Table A1:** Checklist of management in cases of (suspected) mushroom poisoning (data from [30,31,32,33,34]).

	Questions/Measures	Comments
**Patient history**	Where were the mushrooms collected?	Many toxic mushrooms tend to grow in woodlands; growing season and geographic variation may help identify particular mushrooms.
	What type of tree was the mushroom near/on?	*Amanita phalloides* may grow under/near oak or pine trees.
	More than one type of mushroom collected?	
	What did the mushroom look like?	Description might suggest potential for serious toxicity; pictures of mushrooms can be shown.
	How long after ingestion did the symptoms begin?	Symptoms that develop later than six hours after ingestion are often associated with potentially lethal mushroom toxins (exceptions are possible).
	How much was eaten?	Information about the amount may be useful in predicting the course and degree of poisoning.
	Were the mushrooms eaten at more than one meal?	Symptoms may be the result of an earlier rather than the most recent ingestion.
	Were the mushrooms cooked/boiled?	
	Duration and type of mushroom storage?	Most mushroom poisonings are caused by edible mushrooms and are due to incorrect processing after harvesting, e.g., long-term storage of mushroom dishes, mushroom storage in plastic bags.
	Is everyone who ate the mushrooms ill? Is anyone who did not eat the mushrooms ill?	Help differentiate mushroom poisoning from other types of food poisoning.
	Were the mushrooms ingested for recreational purposes?	
	Was any ethanol co-ingested?	Certain mushroom species (e.g., *Coprinus*) can cause a disulfiram-like reaction when ethanol is ingested up to 48–72 h after consumption.
**Physical examination**		Classification of poisoning based upon clinical presentation (e.g., gastroenteritis, liver failure, seizures, cholinergic poisoning, hallucinations, renal failure) helpful in guiding further treatment
**Asservation of mushrooms for analysis**	Whenever possible, samples of all ingested mushrooms should be obtained for potential identification by a trained mycologist. Asservation of remainders of dishes, fresh mushrooms, peeling and waste.	Whole mushrooms are preferred, but identification can be made on parts of the mushroom, especially the cap. Further storage is facilitated by wrapping the mushrooms in wax paper, placing it in a paper bag, and refrigerating the sample. Storage in plastic bags should be avoided.
**Contact with local poison control centre/mycologist**	Consultation with a medical toxicologist/ mycologist is advised to determine likely species ingested based on clinical findings, identification of mushrooms, and specific treatment.	For Switzerland: www.toxinfo.ch, www.vapko.ch
**Laboratory**	Complete blood count, prothrombin time (PT), partial thromboplastin time (PTT), electrolytes, calcium, phosphate, urea nitrogen, creatinine, creatine kinase, liver enzymes, albumin, cholestatic parameters	Additionally in symptomatic patients: - Hepatic failure: blood gas analysis, glucose, lactate, ammonia, lactate dehydrogenase (LDH) - Altered mental status: blood gas analysis, glucose - Cyanosis not responsive to oxygen: methaemoglobin level - Hypoxia: blood gas analysis
**Therapy**	Emergency and supportive measures: - Intravenous hydration, antiemetic therapy - Monitor for 12–24 h for delayed onset symptoms - If mushroom potentially toxic: decontamination with activated charcoal	Specific drugs and antidotes:Seizures: - Seizures: benzodiazepines, pyridoxine - Methemoglobinaemia: methylene blue - Cholinergic syndrome: atropine - Amatoxin-containing mushrooms: silibinin, *N*-acetylcysteine, liver transplant in case of fulminant hepatic failure
**Other**	If amatoxin-poisoning suspected: identification and hospitalisation of the table companions (even if asymptomatic)	

## References

[1] Berger K.J., Guss D.A. (2005). Mycotoxins revisited: Part I. J. Emerg. Med..

[2] Schenk-Jaeger K.M., Rauber-Lüthy C., Bodmer M., Kupferschmidt H., Kullak-Ublick G.A., Ceschi A. (2012). Mushroom poisoning: A study on circumstances of exposure and patterns of toxicity. Eur. J. Int. Med..

[3] Schmutz M., Carron P.N., Yersin B., Trueb L. (2018). Mushroom poisoning: A retrospective study concerning 11-years of admissions in a Swiss Emergency Department. Intern. Emerg. Med..

[4] Eren S.H., Demirel Y., Ugurlu S., Korkmaz I., Aktas C., Guven F.M. (2010). Mushroom poisoning: Retrospective analysis of 294 cases. Clinics.

[5] Lin Y., Wang T. (2004). Mushroom poisoning. Ann. Disaster Med..

[6] Ukwuru M.U., Muritala A., Eze L.U. (2018). Edible and non-edible wild mushrooms: Nutrition, toxicity and strategies for recognition. J. Clin. Nutr. Metab..

[7] U.S. Food & Drug Administration, Center for Food Safety & Applied Nutrition Mushroom Toxins. https://www.med.navy.mil/sites/nmcphc/Documents/nepmu-6/Epidemiology/FDA-Food-Borne-Pathogens/Natural-Toxins/Mushroom-toxins.pdf.

[8] Diaz J.H. (2005). Syndromic diagnosis and management of confirmed mushroom poisonings. Crit. Care Med..

[9] Trueb L., Carron P.N., Saviuc P. (2013). Intoxication par les champignons. Rev. Med. Suisse.

[10] Tox Info Suisse: Annual Report 2016. http://toxinfo.ch/customer/files/638/Tox_JB-2016_140817_DE_ES.pdf.

[11] Pajoumand A., Shadnia S., Efricheh H., Mandegary A., Hassanian-Moghadam H., Abdollahi M. (2005). A retro-spective study of mushroom poisoning in Iran. Hum. Exp. Toxicol..

[12] Unluoglu I., Tayfur M. (2003). Mushroom poisoning: An analysis of the data between 1996 and 2000. Eur. J. Emerg. Med..

[13] Schenk-Jäger K.M., Egli S., Hanimann D., Senn-Irlet B., Kupferschmidt H., Büntgen U. (2016). Introducing Mushroom Fruiting Patterns from the Swiss National Poisons Information Centre. PLoS ONE.

[14] Tox Info Suisse: Annual Report 2016 (Appendix). http://toxinfo.ch/jahresberichte-neu_de.

[15] Ferréol J.-Y. Champignons Cueillis et Utilisés Pour la Consommation Personnelle—Contrôles Effectués en 2014. Swiss Mushroom Control Association VAPKO.

[16] Cervellin G., Comelli I., Rastelli G., Sanchis-Gomar F., Negri F., De Luca C., Lippi G. (2018). Epidemiology and clinics of mushroom poisoning in Northern Italy: A 21-year retrospective analysis. Hum. Exp. Toxicol..

[17] Helfer A.G., Meyer M.R., Michely J.A., Maurer H.H. (2014). Direct analysis of the mushroom poisons α- and β-amanitin in human urine using a novel on-line turbulent flow chromatography mode coupled to liquid chromatography-high resolution-mass spectrometry/mass spectrometry. J. Chromatogr. A.

[18] Index Fungorum. http://www.indexfungorum.org/names/names.asp.

[19] The Portal of the Swiss Government Federal Law: Verordnung des EDI über Lebensmittel Pflanzlicher Herkunft, Pilze und Speisesalz. https://www.admin.ch/opc/de/classified-compilation/20143412/201805010000/817.022.17.pdf.

[20] Swiss Mushroom Control Association Recommendation List (Password-Protected Document). www.vapko.ch.

[21] Swiss Mushroom Control Association List of Poisonous Mushrooms. http://vapko.ch/phocadownload/public/TOUS/Giftpilzliste-Listedeschampignonstoxiques-plusmortels.pdf.

[22] Bon M. (2005). Pareys Buch der Pilze.

[23] Breitenbach J., Kränzlin F. (1984). Fungi of Switzerland.

[24] Breitenbach J., Kränzlin F. (1986). Fungi of Switzerland.

[25] Breitenbach J., Kränzlin F. (1991). Fungi of Switzerland.

[26] Breitenbach J., Kränzlin F. (1994). Fungi of Switzerland.

[27] Breitenbach J., Kränzlin F. (2000). Fungi of Switzerland.

[28] Breitenbach J., Kränzlin F. (2005). Fungi of Switzerland.

[29] Gawlikowski T., Romek M., Satora L. (2015). Edible mushroom-related poisoning: A study on circumstances of mushroom col-lection, transport, and storage. Hum. Exp. Toxicol..

[30] Kupferschmidt H., Rauber-Lüthy C., Schoenenberger R.A., Haefeli W.E., Schifferli J. (2008). Akute Vergiftungen. Internistiche Notfälle.

[31] ©2018 UpToDate Clinical Manifestations and Evaluation of Mushroom Poisoning. https://www.uptodate.com/contents/clinical-manifestations-and-evaluation-of-mushroom-poisoning?search=mushroom&source=search_result&selectedTitle=1~37&usage_type=default&display_rank=1.

[32] Baier J. (1995). Mushrooms and Toadstools: An Illustrated Guide.

[33] ©2018 UpToDate Management of Mushroom Poisoning. https://www.uptodate.com/contents/management-of-mushroom-poisoning?topicRef=13893&source=see_link#H20098225.

[34] Berger K.J., Guss D.A. (2005). Mycotoxins revisited: Part II. J. Emerg. Med..

[35] Parant F., Peltier L., Lardet G., Pulce C., Descotes J., Moulsma M. (2006). Phalloidin syndrome: Role of Elisa-based assay for the detection of alpha- and gamma-amanitins in urine. Preliminary results. Acta Clin. Belg..

[36] Bühlmann Laboratories Amanitin ELISA Product Information. https://www.buhlmannlabs.ch/products-solutions/special-products/amanitin/.

[37] Schenk-Jaeger K., Rauber-Lüthy C., Reichert C., Kupferschmidt H. (2017). Vergiftung mit Knollenblätterpilzen (Amanita phalloides) und Anderen Amatoxin-haltigen Pilzen (Lepiota-, Galerina- und Amanita-Arten). http://toxinfo.ch/customer/files/32/MB-Amanita-D-2017.pdf.

[38] Faulstich H., Jahn W., Wieland T. (1980). Silybin inhibition of amatoxin uptake in the perfused rat liver. Arzneimittelforschung.

[39] Mengs U., Pohl R.T., Mitchell T. (2012). Legalon^®^ SIL: The antidote of choice in patients with acute hepatotoxicity from amatoxin poisoning. Curr. Pharm. Biotechnol..

[40] Poucheret P., Fons F., Doré J.C., Michelot D., Rapior S. (2010). Amatoxin poisoning treatment decision-making: Pharmaco-therapeutic clinical strategy assessment using multi-dimensional multivariate statistic analysis. Toxicon.

[41] Ganzert M., Felgenhauer N., Schuster T., Eyer T., Gourdin C., Zilker T. (2008). Knollenblätterpilzvergiftung. Silibinin und Kombination von Silibinin und Penicillin im Vergleich. Dtsch. Med. Wochenchr..

[42] Giannini L., Vannacci A., Missanelli A., Mastroianni R., Mannaioni P.F., Moroni F., Masini E. (2007). Amatoxin poisoning: A 15-year retrospective analysis and follow-up evaluation of 105 patients. Clin. Toxicol..

